# Healthcare provider perspectives on managing sexually transmitted infections in HIV care settings in Kenya: A qualitative thematic analysis

**DOI:** 10.1371/journal.pmed.1002480

**Published:** 2017-12-27

**Authors:** Kipruto Chesang, Sureyya Hornston, Odylia Muhenje, Teresa Saliku, Joy Mirjahangir, Amanda Viitanen, Helgar Musyoki, Christine Awuor, George Githuka, Naomi Bock

**Affiliations:** 1 Centers for Disease Control and Prevention, Nairobi, Kenya; 2 Centers for Disease Control and Prevention, Atlanta, Georgia, United States of America; 3 University of California, San Francisco, Nairobi, Kenya; 4 University of California, San Francisco, San Francisco, California, United States of America; 5 National AIDS and STD Control Programme, Nairobi, Kenya; Wolrd Health Organization, SWITZERLAND

## Abstract

**Background:**

The burden of sexually transmitted infections (STIs) has been increasing in Kenya, as is the case elsewhere in sub-Saharan Africa, while measures for control and prevention are weak. The objectives of this study were to (1) describe healthcare provider (HCP) knowledge and practices, (2) explore HCP attitudes and beliefs, (3) identify structural and environmental factors affecting STI management, and (4) seek recommendations to improve the STI program in Kenya.

**Methods and findings:**

Using individual in-depth interviews (IDIs), data were obtained from 87 HCPs working in 21 high-volume comprehensive HIV care centers (CCCs) in 7 of Kenya’s 8 regions. Transcript coding was performed through an inductive and iterative process, and the data were analyzed using NVivo 10.0. Overall, HCPs were knowledgeable about STIs, saw STIs as a priority, reported high STI co-infection amongst people living with HIV (PLHIV), and believed STIs in PLHIV facilitate HIV transmission. Most used the syndromic approach for STI management. Condoms and counseling were available in most of the clinics. HCPs believed that having an STI increased stigma in the community, that there was STI antimicrobial drug resistance, and that STIs were not prioritized by the authorities. HCPs had positive attitudes toward managing STIs, but were uncomfortable discussing sexual issues with patients in general, and profoundly for anal sex. The main barriers to the management of STIs reported were low commitment by higher levels of management, few recent STI-focused trainings, high stigma and low community participation, and STI drug stock-outs. Solutions recommended by HCPs included formulation of new STI policies that would increase access, availability, and quality of STI services; integrated STI/HIV management; improved STI training; increased supervision; standardized reporting; and community involvement in STI prevention. The key limitations of our study were that (1) participant experience and how much of their workload was devoted to managing STIs was not considered, (2) some responses may have been subject to recall and social desirability bias, and (3) patients or clients of STI services were not interviewed, and therefore their inputs were not obtained. While considering these limitations, the number and variety of facilities sampled, the mix of staff cadres interviewed, the use of a standardized instrument, and the consistency of responses add strength to our findings.

**Conclusions:**

This study showed that HCPs understood the challenges of, and solutions for, improving the management of STIs in Kenya. Commitment by higher management, training in the management of STIs, measures for reducing stigma, and introducing new policies of STI management should be considered by health authorities in Kenya.

## Abstract 2

In a qualitative thematic analysis, Kipruto C Chesang and colleagues bring the Kenyan STI health care provider's perspective to PLOS Medicine's Collection on STI research.

## Author summary

**Why was this study done?:**

- The objectives of this study were to explore a range of health providers’ professional and personal attributes and structural and environmental factors within clinics, and to use these findings to draw input to the anticipated updating of Kenya’s national sexually transmitted infection (STI) guidelines.

**What did the researchers do and find?:**

- This study was conducted in 21 HIV care and treatment clinics across 7 regions of Kenya, and involved in-depth interviews of healthcare providers working within these clinics.
- A qualitative approach was adopted. The study provided insights into the day-to-day challenges experienced by the health providers and their recommendations for improving STI management.
- The main findings were as follows: Health providers were knowledgeable and prioritized STIs, providers were aware that the number of cases of STIs is high and increasing, most services for STIs except for condom provision were suboptimal, health providers believed STI diagnosis carried high stigma in the community, and community participation in STI prevention and stigma reduction was low.

**What do these findings mean?:**

- The recommendations made by providers to address these challenges were diverse, relating to policy, human resource issues, commodity security, service delivery practices, and quality assurance.
- These recommendations have implications beyond updating guidelines, and suggest a need for more prioritization and resource allocation for STI services. The authors believe that implementation of these recommendations could be key to controlling STIs in Kenya.

## Introduction

Worldwide, more than a million people are infected with a sexually transmitted infection (STI) daily, with the highest burden being in low-income countries [1]. The consequences of STIs go beyond suffering from the infection itself to include adverse health outcomes, as well as social and economic consequences. Adverse health outcomes are varied and can profoundly impact an individual’s physical and mental health. Associated sequelae of STIs include fetal and neonatal deaths, cervical cancer, infertility, increased risk of HIV [2], and low self-esteem [3]. Stigma is an important social consequence that often leads to poor health seeking behavior, denial or minimizing of STI symptoms and consequently delayed treatment, low contact reporting, and further transmission [4]. Stigma can also lead to intimate partner violence, divorce, or family discord if partner notification is not implemented with appropriate sensitivity to interpersonal relationships and social norms [5]. Stigma in STIs stems from the societal intertwining of STIs with moral behavior [6], and worsens when patients with STIs perceive healthcare providers (HCPs) to have a negative attitude toward individuals with STIs [7]. However, health education at the community, patient, and HCP levels, when combined with access to treatment in a supportive and nonjudgmental atmosphere, has been shown to mitigate stigma [8]. Economic consequences of STIs range from direct costs to costs resulting from adverse health outcomes. A systematic review focusing on low- and middle-income countries found that the median treatment cost was US$17.80 per episode, and although it was lower for clinics that were using a syndromic management approach, it was still more than 3 times the average daily income for most people in these countries [9]. Costs associated with medical treatment for adverse health outcomes related to STIs are known to be substantial in industrialized nations, but this has not been well documented in developing country settings [10].The World Bank estimates that 8.9% and 1.5% of the total daily adjusted life years lost due to ill health in developing economies are attributable to STIs, excluding HIV, for women and men, respectively [9].

The burden of STIs in Kenya, as in the rest of sub-Saharan Africa, is high and increasing [11]. In 2012, the overall prevalence of STIs in Kenya amongst individuals aged 15–64 years was estimated at 0.9%. However, the population prevalence for abnormal genital discharge was 6.2% for women and 1.5% for men, while it was 9.8% and 4.6% for women and men living with HIV, respectively [12]. In 2010, a cross-sectional study conducted in Kenya amongst 39 of the 42 largest HIV care clinics, involving 1,063 women and 598 men, revealed that 63.1% of women had at least 1 STI symptom, of which 30.8% were confirmed etiologically. Trichomoniasis was the most prevalent, at 10.5% and 2.8% for women and men participants, respectively [13,14]. These results underscore the fact that STIs are frequent among people living with HIV (PLHIV) and disproportionately affect women. Additionally, because these populations routinely attend HIV care clinics where STI counseling and screening are a part of the routine care package of services, such high STI rates could be an indication of suboptimal service provision. This is distressing news as vaginal HIV-1 shedding has been associated with *Trichomonas vaginalis* co-infection among HIV-infected women, and co-infection may increase the rate of HIV transmission by approximately 2-fold [15]. Moreover, successful treatment of trichomoniasis in women has been shown to reduce vaginal shedding of HIV within 31–87 days [16]. Kenya’s policy for management of STIs is a syndromic approach [17].

In this paper, we document the knowledge and practices, attitudes and beliefs, and perspectives and opinions of HCPs, and the structural and environmental factors within clinics affecting the management of STIs in HIV-infected patients in Kenya. This study adds to previous STI studies in Kenya and other resource-constrained countries, and provides a better-rounded picture of STI diagnosis and management from the HCP perspective that should be considered in the anticipated updating of Kenya’s national STI guidelines [18]. The solutions suggested align with World Health Organization (WHO) guidance for managing STIs as an integral part of comprehensive HIV prevention and care strategies [19].

## Methods

This study was approved for implementation by the Scientific and Ethics Review Unit for the Kenya Medical Research Institute and the US Centers for Disease Control and Prevention (CDC) Division of Global HIV & TB Associate Director for Science and Institutional Review Board.

### Study design

In this qualitative study, we used purposive nonrandom sampling to select HCPs for in-depth interviews (IDIs) from a varied staff mix (e.g., doctors, nurses, and pharmacists) assigned to HIV clinics in Kenya. We will refer to the different staff as cadres. The clinics were drawn from a nationally representative list of 42 large U.S. President’s Emergency Plan for AIDS Relief (PEPFAR)–supported HIV programs in Kenya, previously described by Singa et al. [14]. Of the 42, we selected 21 clinics based firstly on the criteria that each facility had at least 1 clinical staff (nurse, registered clinical officer, or medical officer), 1 pharmacy staff (pharmacist or pharmaceutical technologist), and a clinic manager, and, secondly, to get a representative mix of clinics and HCP types (Table 1). HCPs were then stratified by cadre, sex of HCP, geographic region, level of facility, managing authority, setting, and the clinic patient load using 2010 data. We defined the 7 geographic regions to correspond with the former provinces of Kenya. Level of facility was defined by the Kenya Essential Package of Health, where community, dispensary, health center, primary hospital, secondary hospital, and tertiary hospital are assigned level 1–6, respectively [20,21]. Managing authority was stratified into government (public), faith-based organization (FBO), and non-governmental organization (NGO), and setting was stratified as rural or urban, consistent with the classification used by the Kenya National Bureau of Statistics in the 2009 census [22]: Urban settings were those with city or municipality status, and rural settings were all other categories.

**Table 1:** Health facility characteristics and targeted staff mix for in-depth interviews.

Region	Setting	Adults on ART	Level of facility	Managing Authority	Clinical staff	Manager	Pharmacy staff
Rift Valley	Urban	10,482	6	Public	3	1	1
Rift Valley	Rural	2,794	3	Public	3	1	1
Nairobi	Urban	5,776	6	Public	3	1	1
Nairobi	Urban	1,919	3	Public	3	1	1
Nairobi	Urban	4,565	5	FBO	3	1	1
Nairobi	Urban	2,181	3	Public	3	1	1
Nairobi	Urban	1,269	3	Public	3	1	1
Nairobi	Urban	1,850	3	Public	3	1	1
Coast	Urban	4,335	5	Public	3	1	1
Coast	Urban	3,401	5	NGO	3	1	1
Nyanza	Urban	3,529	5	Public	3	1	1
Nyanza	Rural	4,558	3	Public	3	1	1
Nyanza	Rural	2,282	4	FBO	3	1	1
Nyanza	Urban	5,000	4	Public/NGO	3	1	1
Western	Urban	3,122	4	Public	3	1	1
Western	Urban	1,663	4	Public	3	1	1
Eastern	Urban	2,716	5	Public	3	1	1
Eastern	Urban	1,624	4	Public	3	1	1
Central	Rural	2,738	4	Public	3	1	1
Central	Rural	2,186	5	FBO	3	1	1
Central	Urban	2,109	5	Public	3	1	1

FBO, faith-based organization; NGO, non-governmental organization.

### Setting

The study was conducted in 21 HIV clinics selected within 7 regions of Kenya. These health facilities were of levels 3–6 where HIV/STI services were provided, and also varied on how services were offered. Level 3 facilities, health centers, offer basic curative and preventative health services; level 6, tertiary hospitals, offer specialized and highly skilled medical services; and other levels are in between. In level 6, level 5, and some level 4 health facilities, HIV services were provided in a separate comprehensive HIV care center (CCC) within the hospital, with dedicated staff and units within the clinic offering HIV testing services, consultation services, laboratory services, adherence counseling, tuberculosis care, family planning, and nutrition support. Conversely, most level 3 and some level 4 health facilities had neither dedicated staff nor separate units for clients seeking HIV services, but they made provisions for dedicated clinic days instead. As such, clinic managers in level 6, level 5, and some level 4 facilities were assigned to a dedicated CCC, whereas those of level 3 and some 4 facilities covered all outpatient services within the health facility or were the overall managers for these health facilities.

One-on-one IDIs were conducted in the consultation rooms for clinical staff, in the pharmacy for pharmacy staff, and in the administrative offices for the clinic managers upon ensuring privacy.

### Participants

We targeted a sample size of 105 participants and an equal number of both sexes whenever possible (Table 1). Selection criteria for participants were as follows: (1) employed at one of the HIV care clinics selected for at least 6 months before the study, (2) provided HIV clinical or pharmacy services or served as a clinic manager, (3) provided informed consent, and (4) agreed to be contacted by phone or in person for any clarifications after the interview. However, during the research in the field, some of the eligible participants were traveling, on sick leave, or on vacation, or positions were vacant through the interview period, resulting in 87 interviews being conducted (Table 2). The participants were divided into clinical staff (i.e., medical officers, registered clinical officers, and nurses), pharmacy staff (i.e., pharmaceutical technologists and pharmacists), and clinic managers.

**Table 2:** Characteristics of healthcare providers interviewed (*n =* 87).

Characteristic	Number (%) of staff
**Region**	
Rift Valley	7 (8%)
Nairobi	25 (29%)
Coast	9 (11%)
Nyanza	19 (22%)
Western	10 (12%)
Eastern	7 (8%)
Central	10 (12%)
**Setting**	
Urban	68 (78%)
Rural	19 (22%)
**Healthcare provider cadre**	
Clinical staff	46 (53%)
Pharmacy staff	20 (23%)
Clinic managers	21 (24%)
**Level of facility**	
3	19 (22%)
4	28 (32%)
5	30 (35%)
6	10 (11%)
**Managing authority**	
Public	65 (75%)
FBO	9 (10%)
NGO	13 (15%)
**Sex**	
Female	48 (55%)
Male	34 (39%)
Missing	5 (6%)

FBO, faith-based organization; NGO, non-governmental organization.

### Study procedure, data collection, and management of data

The study was conducted by 2 teams each consisting of a social scientist and a research assistant. A National AIDS and STI Control Programme (NASCOP) representative introduced the team to the health facility staff but did not participate in the actual interviews. NASCOP is a division of the Ministry of Health in Kenya and is charged with oversight and implementation of HIV and STI programs in Kenya.

Prior to the interviews, appointments were made with the potential HCP targeted, and training conducted for research assistants and NASCOP staff on qualitative research methods, ethics, and human participant research principles. The IDI guides were field-tested with 3 HCPs, 1 each from the clinical staff, pharmacy staff, and clinic manager categories, who confirmed the appropriateness of the questions. Each session lasted 0.5‒1 hour.

We conducted the interviews from June 19, 2012, to August 31, 2012. Interviews were conducted in English after participants provided written informed consent and contact information. In a few instances where HCPs interjected with Kiswahili language phrases to express their opinions better, the research assistants, who were well versed in Kiswahili, provided the translation as necessary. The NASCOP staff were not involved in data collection, to safeguard the confidentiality of participants and to avoid participant–supervisor bias.

Using separate IDI guides that had open-ended questions for clinical staff, pharmacy staff, and clinic managers, data were collected via semi-structured IDIs, audio recorded, and observation notes made. The domains of interest were knowledge and practices, attitudes and beliefs, structural and environmental factors affecting STI management, facilitators and barriers to management of STIs, and perspectives of HCPs on the management of STIs. We started with broad questions, and if we could not elicit spontaneous responses to specific aspects, we followed with probes. For instance in asking about practices, we first asked how STIs were treated in their clinic, and then followed with a probe asking specifically if they had and used STI guidelines or standard operating procedures if that information was not part of the initial answer. We used the Kenya guide *Integrating the Management of STIs/RTIs into Reproductive Health Services—Pocket Handbook for Health Providers* [23] and the WHO *Guidelines for the Management of Sexually Transmitted Infections* [24] to define standards for knowledge and practices. Because we wanted to understand HCP perspectives on how each HCP individually prioritizes STI management, we included a descriptive rating scale of 1–10, where 10 is highest and 1 is lowest, in the IDI guides.

The audio records were transcribed verbatim to produce texts of the interviews in Word, with observational notes included. Quality control procedures for interview transcription were as follows: (1) we limited transcription to 3 interviews per day per transcriber to ensure thoroughness and (2) the social scientist reviewed the transcribed scripts on a daily basis and discussed any gaps or corrections. Overall, most of the transcriptions passed the quality control check with no amendments.

Each transcribed script was given a unique identifier composed of region, type of facility, cadre, setting, managing authority, and the sex of HCP. All the transcribed scripts and audio tapes were securely stored, and, as per the study protocol, audio tapes were destroyed 2 weeks after the transcription and quality control checks had been completed. No transcribed scripts contained personal identifiers.

### Data analysis

An inductive, iterative process was used in the analysis, to explore broad themes, commonalities, and constructs within the domains of interest.

Reviewers developed a preliminary codebook using 20 interviews through an independent review of 5 different transcripts each by 4 reviewers. The codebook consisted of the codes, sub-codes, definitions, and guidelines on when to use and when not to apply. Once the codebook was finalized, coding of the transcripts was done by 4 independent coders. After the coding of 2 more transcripts, to make a total of 22, a preliminary analysis was done to determine if all themes were captured well and if there were any new emerging themes, and to check for saturation of themes. Review of this preliminary analysis provided insight on merging some themes and ensuring a balance of information from all cadres, facility levels, regions, settings, managing authorities, and sexes. We determined saturation using guidance previously described by Morse [25,26] and Guest et al. [27]. Saturation was reached after coding a total of 61 transcripts, and these transcripts were used for analysis using NVivo 10.0 (QSR International). All the 22 transcripts for the FBOs and NGOs and 39 for the publicly managed facilities were coded. Certain quotes were selected to highlight the important points made by the participants. The remaining 26 interview transcripts were reviewed after analysis; review involved reading through each script, confirming that all themes had been captured, reinforcing themes already identified, and selecting quotes if better phrased than those from the initial analysis.

We analyzed the collective priority of STIs among HCPs using the 1–10 scale described above after establishing a range that would cover 90% of HCP responses from 10, the highest, and downwards to the 90% mark, and then further analyzed the reasons based on the top 90% and the remaining 10%. To interpret our findings on knowledge and practices, we used standards set in the guidelines described above [23,24].

## Results

### Characteristics of the study participants

Of the 87 participants, HCPs in the Nairobi and Nyanza regions were proportionately more due to the number of clinics selected (Table 2), but the distribution was proportional to Kenya’s HIV burden pattern. The majority of participants were in urban settings, and about half were clinical staff, while pharmacy staff and clinic managers each comprised roughly one-quarter. Over half of the participants were in level 3 and 4 health facilities, and 75% were based in publicly managed health facilities, while the remaining 25% were in FBO, and NGO managed health facilities; this was proportionate with the PEPFAR pattern of support in Kenya. Although the intent was to have both sexes represented equally, 55% of the participants were female, 39% were male, and 6% did not have sex specified.

Overall, despite the extensive variation of our sample, our analysis showed broad homogeneity in findings across cadres, facility levels, regions, settings, managing authorities, and sex of HCP, except for access to STI drugs. NGO facilities had better STI drug provision compared to both FBO and public facilities, where stock-outs were frequent.

### Knowledge and practices

#### Knowledge of STI management

HCPs had good technical knowledge of STIs. They understood the relationship between HIV and STIs, modes of transmission, symptoms and signs, major causative agents, complications, prevention methods, and the importance of treatment compliance, condom provision, counseling, contact tracing, and reporting requirements. On the prioritizing of STIs in their work, ranking from the highest, 90% of HCPs gave a score of 5–10. The reasons given for high prioritization of STIs were (1) STIs being seen as a major health problem and as one of the top 10 most common diseases, (2) understanding that STI transmission mirrors HIV transmission, and (3) the increasing antimicrobial drug resistance to some STI agents. Those who placed a low priority on STIs cited that this low priority was based on STIs receiving limited attention from higher levels of management, and not their personal views.

Respondents viewed co-infection with STIs among patients with HIV to be very high. Across clinics, providers reported that the prevalence of STIs was higher among women, teens, and young adults. Providers understood that women with STIs were more likely to be asymptomatic than men, but also mentioned that women were more willing to disclose their symptoms due to the multiple contacts they made with the healthcare system, such as during antenatal, postnatal, and family planning consultations. Women were thought to have higher prevalence of STIs; this was attributed to women not knowing when they were infected and thereby delaying treatment, their inability to negotiate condom use with partners, being ashamed, thinking any vaginal discharge was normal, and having inadequate information on STIs. In addition, providers thought that men usually sought treatment elsewhere, such as pharmacies and private facilities. The majority of HCPs also thought STI incidence was increasing.

Genital discharge, warts, and genital ulcer disease (GUD) were cited as the most common STIs, and in 1 extreme case, a clinic manager estimated that half of the clients seen in that clinic every day also had an STI, particularly GUD.

Moderator: You see 80 patients a day, how many would you say have an STI?

HCP: About a half of them.

Moderator: Half of them per day, quite high.

HCP: Yes, but maybe it’s not STI like you can say it’s urethral [discharge] but GUD especially when they are on 3rd or 4th [stage] in terms of their [WHO staging] status. Maybe CD4 is very low and that is when we start seeing a lot of GUD. [Male, Clinic Manager, Level 4, Public]

Most HCPs reported managing STIs using the syndromic approach, except in the management of GUD, for which they requested syphilis testing. In many instances, HCPs would also use the etiologic approach when the patient was able to pay for laboratory services. In addition, most HCPs were unfamiliar with anal STI diagnosis and did not have confidence with the algorithm used at the time of the study.

Sometimes we take you to the lab when the client himself says I have the money that is needed in the laboratory. And when the client tells you I don’t have anything, you will be forced to treat syndromically, yes. [Male, Clinic Manager, Level 3, Public]

#### Screening practices

Screening practices were diverse at both healthcare provider and health facility levels. Some clinics conducted screening routinely at every visit, others twice a year, while others reserved it only for new patients. There was no standard screening procedure. Most providers only inquired about genital symptoms verbally and if the response was “no,” they did not further investigate. Even this verbal screening was not universal as some providers omitted it due to high HIV-service-related workload, while some thought patients would not disclose due to stigma. Physical examination followed for those who disclosed symptoms, and laboratory blood testing was usually conducted for those with GUD to rule out syphilis. Most HCPs described a more comprehensive approach for first-time clients, survivors of rape, antenatal women, women seeking family planning services, and those who self-identified as female sex workers, for whom screening by physical examinations was conducted. However, anal screening was not usually done.

Moderator: Have you considered finding out if your patient has anal symptoms resulting from STIs?

HCP: Unless they tell us they have a problem, routinely no.

Moderator: Any reason why that consideration has not been thought about?

HCP: Yeah because they’ve not complained. I don’t know, it has not been implemented. [Female, Clinical Staff, Level 5, Public]

Moderator: Have you considered finding out if your patient has anal symptoms resulting from an STI?

HCP: Not really.

Moderator: Do you do that at all?

HCP: It’s not done. [Male, Clinical Staff, Level 4, FBO]

In summary, screening was negatively affected by the lower emphasis placed on STIs compared to HIV, lack of standard operating procedures, HCPs’ discomfort in discussing sexual issues, and heavy patient workload. HCPs reported being unfamiliar with how to take clinical history for and manage anal STIs.

#### Syndromic STI management and contract tracing

Most HCPs used syndromic management, even though many had no access to the treatment algorithm and STI guidelines. Providers cited the benefits of syndromic management as (1) availing treatment quickly, (2) upholding confidentiality and reducing stigma, (3) not losing patients through referral to the laboratory, and (4) avoiding the added laboratory costs. While appreciating the extra resource requirements for etiologic diagnosis, some HCPs would prefer this approach because it offers improved specificity in diagnosis, better management of recurrent infection, elimination of unnecessary medications, and monitoring for drug resistance. Expedited partner therapy, i.e., giving patients medication for their partners as well as themselves, was generally not done, and in the few instances when it was done, the clinicians pointed out that it was not standard practice as it still missed the partner counseling component.

The use of contact tracing was diverse among clinics; some conducted routine home follow-ups or calls or partner referral, while others could not do any of these owing to staff shortages. HCPs reported that partner referrals were often unsuccessful.

I believe the partner should also be treated whether symptomatic or asymptomatic. But explaining that to patients for them to understand is quite a challenge.… They say, “No, we can't do that.” They think this and this.… They say, “Ah, you are sick, it’s only you. Me, am not.” It’s rare for them to come, only the few concerned ones come. [Female, Clinical Staff, Level 5, Public]

#### Counseling and condom provision

Most HCPs considered counseling to be important. Some providers were systematic and explained to their patients about STIs, how STIs are sexually acquired and transmitted, treatment options, prevention, and how to avoid recurrence. Even so, HCPs still thought counseling at the individual level was inadequate, and they recommended combining it with community education.

Most HCPs provided condoms even when patients did not request them. In addition, many health facilities had condom dispensers strategically placed for patients to take condoms on their own. Condom counseling was frequently practiced, using appropriate demonstration tools.

[We provide condoms] as often as required. Ideally we’re supposed to give condoms to each client who is sexually active.… For those who don’t know how to use it, we usually send them…there is a room for demonstration purposes. In fact sometimes we tell them, “take” even if they don’t want because we know they may need it. So we provide them. [Female, Clinical Staff, Level 5, Public]

#### STI reporting practices

Most HCPs were unaware of a specific STI reporting tool, and some thought the tool was data extracted from the Positive Health, Dignity and Prevention checklist question found in the HIV patient charts [28]. Contact tracing reporting was limited to processes developed by individual facilities.

We don’t report because we have no reporting tools for STIs so we just see the patients and we leave, we don’t document unless in their files and their books but we’ve no tools for reporting STIs. [Female, Clinical Staff, Level 4, Public]

Consequently, HCPs suggested that reporting tools for STIs should be made available, data generated from reports should be used to inform facility decisions, and STI and HIV reports should be integrated so as to attract the interest of higher authorities.

As long as they [higher management] will be receiving reports on HIV, he or she should also receive report on STIs. [Male, Pharmacy Staff, Level 3, Public]

#### Support supervision and training

Support supervision at regional and national levels was minimal. Although a few facilities had regular discussions on STIs during meetings and scheduled continuing medical education, most HCPs had not received in-service trainings. As a result, HCPs reported lack of confidence, doubted their competence, and reported that STI treatment prescribing practices were inconsistent between clinicians.

Moderator: How often do you get support supervision for management of STIs?

HCP: It has actually taken a longer time, because since I came to this facility in 2009 we’ve never had support supervision for management of STIs. [Male, Clinical Staff, Level 4, Public]

On training, HCPs reported that they lacked capacity in STI management including broader issues of sex and sexuality, and that going by the method then used in selecting HCPs for off-site residential in-service trainings, no criteria were being used, sometimes resulting in training being allocated inappropriately, such as to HCPs trained before in the same course.

The one who was trained will be trained [again] next time. So it’s good you make sure that this person was trained last time so that next time you get another person. [Female, Clinical Staff, Level 4, Public]

The only thing that I want to be frank and say is that we really need training on this about sex and sexuality. It’s not very easy for many of us to bring out the wordings around sex.… In our clinic we have those people who have orientations we don’t want to talk about and sometime it’s not easy for many of the clinicians to know who they are. [Female, Clinic Manager, Level 5, Public]

To solve supervision and training challenges, HCPs recommended that (1) all levels of management should improve support supervision for STI services, (2) NASCOP should develop training curriculum options for residential, on-the-job, and continuous medical education, (3) NASCOP should define a fair and impartial training selection process (i.e., selecting only the relevant individuals and giving equal opportunity to all HCPs), (4) NASCOP should put in place an annual training plan to mitigate the effects of high staff turnover, and (5) HIV training and mentorship programs should be integrated with STI training and should specifically include information on anal sex, sexuality, and STI prevention counseling.

#### STI/HIV service integration

Most HCPs cited that they would prefer integrating STI and HIV services in the CCC because STIs were common amongst their clients. During the survey, most HCPs reported that STI and HIV services were not integrated. Some HCPs in busy clinics would prefer integration to be implemented through a separate STI clinic within the CCC, while the majority would prefer that both STI and HIV services be addressed together during the same consultation. Providers attributed lack of integration to vertical HIV programming, while at the same time blaming the rising HIV prevalence on the lack of integration, which resulted in poor management of STIs and increased transmission of HIV. The maternal-child health (MCH) service delivery model of integration was a solution that was suggested frequently.

Integrate the management…just like it’s in one place. Like in my setting it’s done in the MCH so if a patient doesn’t pass through MCH the patient will have to pass through this. So…decentralize to every unit not just the MCH but the patient support center, the specialized clinics, the wards, and I think that would capture STI [Female, Clinical Staff, Level 5, Public]

The advantages of integrating STI and HIV services mentioned were stigma reduction (particularly among men), saving time, improving patient management through capturing better quality sexual history, early detection of STIs and better contact tracing, improving confidentiality and HCP–patient relationship, easing access by providing a one-stop service, and providing a holistic package of services. Providers also said that integration supports the health system by enabling availability of appropriate equipment for screening, reducing missed opportunities for treating STIs, being less costly overall due to shared resources, having fewer STI drug stock-outs through integrated management, and improving reporting. Barriers limiting integration were noted to be the shifting national policies on integration, the conflict of charging for STI services while HIV services are free, heavy workload, inadequate knowledge on STI management, and high staff turnover. In contrast to the opinion of some HCPs, some HCPs thought integration would increase stigma as some patients with STIs might not want to be associated with HIV through STI services being co-located with HIV services; they also thought that it would make contact tracing difficult since sexual partners would further shy away. Some providers also viewed integration negatively as it would increase workload and the time spent per patient. To facilitate integration, providers recommended alignment of new programs with ongoing programs by donor agencies.

### Beliefs and attitudes

Most providers believed that STI management was important because STI burden was high among HIV patients, STIs further suppressed HIV patients’ immunity, and untreated STIs resulted in complications or cases becoming chronic. Moreover, HCPs believed that STIs facilitated HIV transmission and that by controlling STIs, HIV would also be controlled. Providers also believed that the prolonged shortage of STI medications or ineffective STI medications contributed to the rising burden of STIs in the community. Although most providers used an STI syndromic approach, most believed there was growing drug resistance to the current STI medications.

Somebody may be presenting with the same complaints even after treatment. We use syndromic approach and even ensured compliance.… We’ve even treated the partner and did everything else, but it’s still recurrent. It’s a major challenge. [Female, Clinical staff, Level 5, Public]

Across all clinics and regions, providers believed that anal STIs were not a problem in their geographic area; hence, HCPs did not feel the need to pay any special attention to this matter. Providers believed that counseling, condom education, and contact tracing were a part of their responsibilities, although contact tracings were not being optimally conducted. Some HCPs also thought that other HCPs minimized STIs and that STIs required increased awareness.

You know, we may be health workers, but some may not take it as something big. It’s like kitu ya kawaida [something normal]. So if there is that awareness,…education about its importance, maybe people will be aware. [Female, Clinical Staff, Level 5, Public]

Providing health education on STIs at the community level was considered critical by most HCPs as the community was thought to believe that people with STIs also had HIV, as both were connected through sexual acquisition. Providers thought that knowledge on the correct use of condoms was limited within the community and that having multiple sexual partners among PLHIV was rampant because they felt healthy after starting antiretroviral treatment. HCPs believed that stigma was high for both HIV and STIs independently, but were equally divided on the subject of whether STIs or HIV carried more stigma, with some citing less stigma for STIs because most are curable. While some HCPs thought HIV was more acceptable due to sustained sensitization, STIs were associated with immorality as the community viewed STI acquisition to be possible only through sexual intercourse. HCPs reported that patients themselves harbored high self-stigma and guilt for not heeding HIV prevention measures taught during routine HIV care consultations.

There are [sic] still some stigma within the community pertaining STIs, HIV included, and at times people withhold important information or just give you half information and leave something concerning STI. In this case, people are informed that protected sex is a requirement in HIV care and treatment, and therefore if he or she discloses that he/she might be having an STI it’s a clear indication that he/she doesn’t engage in protected sex. So for that, they choose to hide. [Male, Clinical Staff, Level 4, FBO]

Stigma is further compounded by some cultural beliefs concerning STIs. A HCP from the Coast region cited a common belief in the region when she stated that STIs are seen as a punishment for having sex with an underage person.

Umetembea na rafiki ya mwanako ndiye amekuharibu umepata hiyo. [You had intercourse with the friend of your child who afflicted you and you got that.] [Female, Clinic Manager, Level 5, Public]

Providers thought stigma led to nondisclosure at the CCC and to patients’ sexual partners, and consequently resulted in delayed treatment of STIs and patients infecting or reinfecting their partners. Providers also thought patients feared that HCPs could breach confidentiality and leak information of their condition to others in the community. It was reported that patients felt more comfortable with clinicians with whom they were familiar from previous visits and who were the same sex as the patient, but they were not comfortable with providers with whom they interacted socially. HCPs thought that female patients were more self-assured, while males feared coming to the clinics and were more likely to seek treatment at private pharmacies.

Most of the times we see more women presenting with STIs. And this could be because they actually open up and come. The men fear to come. [Male, Pharmacy Staff, Level 4, Public]

Local customs and other cultural beliefs related to traditional male masculinity gender roles were cited as some of the barriers preventing men from accompanying their spouses during contact tracing and from using condoms. Even though women and adolescents were the majority of patients, nonetheless, they still waited until their medical conditions were severe to seek treatment. In addition, dislike of taking medicines in general and the belief by some patients that HIV therapy also treats STIs contributed to the delay.

But I think they [men] really need more health education because we are even encouraging, other than the STIs, for expectant mothers to be accompanied of their partners. But it is still a problem. I think it could be due to the customs of the area and other cultural issues. [Male, Clinical Staff, Level 4, Public]

Some HCPs believed that NASCOP was somehow committed to STI programming because of the initiative to conduct this study, the inclusion of STIs in the HIV/AIDS management protocols, and the combining of both STI and HIV in mass media campaign messages. However, HCPs in most clinics believed higher levels of management showed limited commitment due to inadequate support supervision of the STI program. Moreover, HCPs considered the current donor dependence for staffing at CCCs as unsustainable. HCPs also expected NASCOP to fulfill other responsibilities, such as formulating and disseminating policies/guidelines and providing trainings and a steady supply of drugs, and they questioned whether NASCOP was meeting these expectations. Although some providers attributed the perceived low commitment to the increasing threats of new diseases, many thought it was because of the increased attention on HIV programming at the expense of STIs.

The STIs have been overcome by HIV as everyone is talking about HIV. But you’ll get very few people talking of STIs which are still there. [Female, Pharmacy Staff, Level 5, Public]

Overall, HCPs had a positive attitude and concern for PLHIV co-infected with STIs. Most were empathetic, observing that STIs are a rising societal problem that goes beyond PLHIV and specific sex and age groups, and that negative HCP attitudes yield undesirable responses from the patients. However, even with those considerations, some HCPs still blamed patients with STIs for not using condoms, which is part of HIV prevention education.

You’ll find a clinician will not understand why this patient has an STI yet they are [supposed to be] using condoms [and will ask the patient] “why then did you get the STI?” Those are the kind of questions our patients are running away from. So I don’t know how we should handle them, because then may be I’ve been so persistent in telling our patients to use condoms.… [Female, Pharmacy Staff, Level 5, Public]

### Structural and environmental factors affecting STI management

Across all facilities, HCPs were unaware of a specific STI policy, but knew about policies relating to HIV services. As such, some facilities integrated STI services with HIV services, while others referred co-infected patients to STI clinics. Even among those facilities that integrated services at the CCC, only a few stocked STI drugs within the CCC, with some reporting inconsistent availability, while the majority referred patients to the main hospital pharmacy, which was often far and inconvenient. Although most HCPs said they would prefer STI treatment to be free, only in a few facilities did respondents cite that it was free for STI/HIV co-infected patients. Furthermore, in facilities where patients had to pay, drugs such as metronidazole, benzathine penicillin, doxycycline, amoxicillin, procaine penicillin, and norfloxacin were mostly prescribed, while acyclovir and antifungals were generally unavailable. Some HCPs prescribed drugs based on availability rather than the STI algorithm. HCPs suggested the reintroduction of the STI kit policy (fixed set of combination medicines for STI syndromic management prepacked for multiple patients before distribution to health facilities) or medicines that are prepackaged for individual patients according to the 4 main STI syndromes, so as to improve the availability of STI drugs, minimize diversion, and standardize treatment regimens.

It’s like the way the TB program, they have their own drugs, patients packs.… So once we’ve got a client with STI and we know this is the pack for this client [Male, Clinic Manager, Level 4, Public]

HCPs also recommended the introduction of an STI policy with the following included: (1) free STI services in public health institutions, (2) integration of STI and HIV services, (3) STI medications available over the counter in private pharmacies and dispensed by pharmacy staff trained in STI syndromic management, (4) establishment of an STI referral system, and (5) STI services linked at the community level.

Although guidelines and/or job aides were available in half of the facilities, HCPs said that these tools required updating. Most facilities had adequate waiting rooms, but inadequate consultation room spaces, thus compromising patient confidentiality and limiting privacy both for physical examinations and counseling. Privacy was mentioned as important in empowering patients with tailored health education sessions, prevention measures, and stigma reduction. The majority of the facilities had an examination couch, but lacked equipment, such as speculums, appropriate lighting sources, and equipment for sterilization, which resulted in referrals in cases of female pelvic examinations to other hospital units. For those HCPs who preferred etiologic diagnosis of STI, laboratory supplies and equipment were limited. As such, HCPs proposed that (1) STI guidelines, charts, and algorithms be updated based on effective treatment options; (2) the clinical environment for STI service delivery be made conducive to providing patient confidentiality and privacy; and (3) specific health facilities identified for etiologic diagnosis, e.g., referral sites, have appropriate capacity.

Providers reported that staff shortages, stress, high staff turnover, and burnout were frequent. As a result, HCPs felt inadequate in providing prevention counseling and client-tailored services, leading to many referrals of patients suspected with STIs. Although all clinics had appropriate clinical staff to manage STI patients, community linkages were poor, which compromised STI health education, stigma reduction, and contact tracing at the community level.

You also need community involvement.… If you get more than three STI cases in a day, it means there are some that you have missed in the community who may have not come for the treatment. So…you also need to organize for community outreach.… You also involve the stakeholders so that you can capture those who are still having the stigma. [Male, Clinical Manager, Level 4, FBO]

Solutions recommended by HCPs were increasing staffing at the CCC and better triaging, and community health workers being trained and equipped to implement STI prevention, stigma reduction, early seeking of treatment, and partner disclosure/referral within the community.

## Discussion

Our study found that HCPs were encountering many STIs within the PLHIV population, but were constrained in their ability to respond despite considering it as a high priority. The standards of managing STIs were limited due to inadequate supervision, inadequate reporting, and the cessation of specific STI trainings that were conducted in the past. Furthermore, due to inadequate prevention approaches, particularly compliance with medication doses and contact tracing, STI cases and stigma continued to rise.

Overall, HCPs were knowledgeable, and clearly identified challenges and solutions for improving STI management. Integrating STI management with HIV management, training and mentorship programs, availability of guidelines and job aides, and free STI medications were the main solutions suggested by HCPs. Other solutions included improving supervision and standardizing reporting, community involvement in prevention, service provision by same-sex providers, and more commitment to STI services by higher levels of authority. The solutions offered will be considered during the anticipated updating of the national STI guidelines scheduled to be completed in 2018. The HCP preference for integration of STI and HIV services is consistent with WHO recommendations for developing a viable public health system [29]. This approach is further supported by the successful experiences in STI and family planning integration programs, where HCPs found that the “one-stop” service system was more convenient, improved client satisfaction and access to services, and reduced stigma in a cost-effective manner [30,31]. However, to facilitate a good integration, HCPs should be trained and equipped with the right skills. This is especially important in Kenya, where the majority of providers are nurses who have been task-shifted to provide HIV services but have not been equipped with the skills for providing STI services. Several studies have shown that HCPs with adequate training also have positive attitudes and higher clinical practice scores [32–34]. This agrees with our findings, which showed that although HCPs portrayed a positive attitude towards PLHIV with STIs, it was not reflected in their practice as most did not screen for STIs despite their belief that many PLHIV were in multiple sexual relationships. From ethical and public health points of view, the high level of STIs within the PLHIV population warrants integration of STI and HIV services.

We also observed gaps in provider–patient communication that insufficient training on STIs and running busy CCCs would not wholly explain. We attribute the poor communication to inadequate skills in interpersonal communication and the art of shared HCP–patient decision-making, and to stigma. Consideration of provider–patient communication was first documented during the times of ancient Egypt, and the concept further developed during the Greek enlightenment (5th century BC), with Hippocrates suggesting provider communication may influence patients’ health [35,36]. Fong and Longnecker further describe effective doctor–patient communication as the heart and art of medicine in the delivery of healthcare [37]. Rather than being attentive, warm, listening, empathizing, personal, and using open-ended questions as expected in establishing interpersonal relationships [36,37], HCPs in our study often made statements implying that the burden of communication was on the patient, such as “he/she did not say…” or “she/he did not complain of….” It is also possible that providers avoided asking patients about STIs due to the fear of STI stigma interfering with the HCP–patient interpersonal relationship, and instead preferred to concentrate on HIV management, the primary reason, perhaps in the HCP’s view, for the patient seeking consultation. This avoidance behavior may further be discerned by patients through observations of HCP nonverbal communication, leading to suppression of disclosure and delayed treatment and adversely impacting recovery. Additionally, the perceived associations of unprotected sex and HIV with immorality may have reduced HCPs’ and patients’ inclination to discuss STI risks and symptoms. This is a disjunction fallacy, especially when providing care for PLHIV, because the true associations between HIV, unprotected sex, and STIs actually increase the probability of STIs in this patient population. Tversky and Kahneman observed this type of disjunction fallacy among physicians [38], and Young et al. demonstrated that people actively seek to avoid a stigmatized identity, even at the expense of their actual health, and evade unwelcome conclusions when faced with the possibility of a negative health outcome [6]. HCPs caring for PLHIV may have also implicitly circumvented the possibility of occasioning “double devaluation” feelings among their patients. Alternatively, patients may have decided to protect themselves from stigma [39], and become evasive in discussing STI symptoms, thus making it difficult for HCPs to make further inquiries.

Our study was confined to HCPs who worked in CCCs treating PLHIV, but the revelation of a high burden of STIs among PLHIV could also be a critical indicator that STIs are also high in the general population, given that Kenya’s HIV epidemic is not limited to certain populations, but rather is driven by the general population. For instance, the Kenya AIDS Indicator Survey 2012 noted that almost two-thirds of sexually active women and men had never been tested for HIV with their last sexual partner and that one-third of HIV-infected persons had not disclosed their HIV-positive status to their last sexual partner [12]. Additionally, a population-based study in Malawi within an area with an HIV prevalence of about 10% showed multiple chains of connections in a sexual network, leading to half of all sexually active respondents being linked together in a giant network [40]. Although HIV care and treatment clinics are the logical settings to provide comprehensive services, it is critical that STI services are integrated with other services at health facilities where both HIV-infected and -uninfected males and females can have easy and free access.

Availability of free STI treatment was one of the highly recommended points by HCPs. Due to frequent stock-outs of STI medications, HCPs were sometimes forced to prescribe what was available at the health facility pharmacy, such as amoxicillin and procaine penicillin, even though these drugs were outside the guidelines. HCPs’ recommendation to have STI medications specifically packaged in accordance to STI syndromes would improve compliance to the treatment algorithm and reduce stock-outs or diversion of STI medications to elsewhere. To bring down STI prevalence quickly, expedited partner therapy with emphasis on regular partners, including those in a union, should be implemented in line with the WHO Global Health Sector Strategy on Sexually Transmitted Infections 2016–2021 [41]. Enabling retail pharmacies to prescribe STI medications over the counter would perhaps be most impactful. A previous study in Kenya showed that retail pharmacies are often the first and sometimes the only place for people to get treatment for STIs, but this may result in inappropriate treatment [42]. Such a strategy of using retail pharmacies should therefore start with training and certification of pharmacists in these outlets, and be followed by continued monitoring of the quality of STI services.

We also propose that since the majority of providers in Kenya are registered clinical officers and nurses [43], these cadres should be prioritized for training using an expanded curriculum on STIs that includes stigma, sexuality, and anal sex. Our findings showed a dearth of information on and discomfort about anal STIs among HCPs, leading to neglect and perhaps mismanagement of patients with anal STIs. Training should therefore be combined with broader understanding of anal sex, while paying attention to the sensitivities surrounding this subject. Further, because of concerns by HCPs on treatment failures and emerging resistance of STIs to the antimicrobials then in use, STI referral sites should be equipped with the capacity, including expertise, to implement etiologic management of STIs and routinely report any resistance profiles. Improvements will also depend on adapting new STI policies and guidance from WHO and the CDC, in addition to those already identified by HCPs. Another key finding of this study is that the HCPs who identified STIs as a low priority did so because of their perception of the low level of commitment by higher management authorities, although they personally thought otherwise. This finding is supported by the theory of reasoned action, which posits that attitudes towards a behavior are subjective to the norms of one’s peers [44]. Higher levels of authority should therefore consider improving their commitment to STI management.

Our study is subject to several limitations. First, the selection of clinics was not random and, as such, may not be representative of all clinics in Kenya. Furthermore, because selection was based on PEPFAR-supported sites, public and FBO-supported health facilities may have been overrepresented. Second, participant selection was done to ensure inclusion of all cadres and was based on convenience. In addition, we did not collect information on a number of factors that may have influenced participants’ responses, such as experience and amount of time they spent managing STIs. This is likely to have impacted our findings of knowledge of STI management. Third, some responses may have been subject to recall and social desirability bias. Fourth, although the overall objective of the study was to improve the management of STIs, the patients and clients of STI services were not interviewed, and therefore their inputs were not obtained. Fifth, due to security concerns at the time, the North Eastern region of Kenya was not included. While such limitations are important to consider, the number and variety of facilities sampled, the mix of cadres interviewed, the use of a standardized instrument, and the consistency of responses add strength to our findings.

In conclusion, using a qualitative design, this study explored a range of HCP professional and personal attributes, and structural and environmental factors within CCCs that could influence STI services for STI/HIV co-infected patients. The study has provided insights into the day-to-day challenges experienced by HCPs and their recommendations. These recommendations have implications beyond updating guidelines, to more prioritization and resource allocation for STI services. We believe that urgent implementation of these recommendations could be key to controlling STIs in Kenya.

## Abbreviations

CCC: comprehensive HIV care center
CDC: US Centers for Disease Control and Prevention
FBO: faith-based organization
GUD: genital ulcer disease
HCP: healthcare provider
IDI: in-depth interview
NASCOP: National AIDS and STI Control Programme
NGO: non-governmental organization
PEPFAR: U.S. President’s Emergency Plan for AIDS Relief
PLHIV: people living with HIV
STI: sexually transmitted infection
WHO: World Health Organization
